# Dynamics of Fat Oxidation from Sitting at Rest to Light Exercise in Inactive Young Humans

**DOI:** 10.3390/metabo11060334

**Published:** 2021-05-24

**Authors:** Julie Calonne, Elie-Jacques Fares, Jean-Pierre Montani, Yves Schutz, Abdul Dulloo, Laurie Isacco

**Affiliations:** 1 Department of Endocrinology, Metabolism & Cardiovascular System, Faculty of Science & Medicine, University of Fribourg, 1700 Fribourg, Switzerland; calonne.julie89@gmail.com (J.C.); ef08@aub.edu.lb (E.-J.F.); jean-pierre.montani@unifr.ch (J.-P.M.); yves.schutz@unifr.ch (Y.S.); abdul.dulloo@unifr.ch (A.D.); 2 Department of Nutrition and Food Science, American University of Beirut, Beirut 11072020, Lebanon; 3 AME2P laboratory (AME2P, EA 3533), Clermont Auvergne University, CRNH Auvergne, 63001 Clermont-Ferrand, France

**Keywords:** obesity, physical activity, energy expenditure, sedentary

## Abstract

Societal erosion of daily life low-level physical activity has had a great influence on the obesity epidemic. Given that low fat oxidation is also a risk factor for obesity, we investigated, in a repeated measures design, the dynamics of fat oxidation from a resting state to a light-intensity leg cycling exercise (0–50 watts) in inactive, healthy young adults. Using indirect calorimetry, energy expenditure and the respiratory quotient (RQ) were assessed in a sitting posture at rest and during a cycling exercise in 35 subjects (20 women). The rate of perceived exhaustion (RPE) was assessed using the Borg Scale. During graded leg cycling, the mean RPE did not exceed values corresponding to the exercise being perceived as ‘light’. However, analysis of individual data at 50 watts revealed two distinct subgroups among the subjects: those having RPE values corresponding to the exercise being perceived as ‘very light to light’ and showing no increase in RQ relative to resting levels, as opposed to an increase in RQ in those who perceived the exercise as being ‘somewhat hard to hard’ (*p* < 0.001). Our study in inactive individuals showing that high fat oxidation was maintained during ‘light-perceived’ physical activity reinforced the potential importance of light physical activity in the prevention of obesity.

## 1. Introduction

The matching of energy expenditure (EE) to energy intake is a fundamental feature of the long-term regulation of body weight [1,2]. Traditionally, the importance of physical activity in this matching has centred upon moderate- to vigorous-intensity exercises, and a lack of such structured physical activities has been commonly implicated in the development of obesity and other health risks [3]. While such structured exercises are beneficial in the prevention and management of cardiometabolic diseases, individuals most often do not closely adhere to training programs, specifically outside medically-supervised settings, mainly due to their monotony and difficulty and/or to the subjects’ motivation and way of life. Thus, long-term benefits remain elusive, and it is vital to develop strategies that simultaneously support health and fit with an individual’s abilities and possibilities to increase adherence and maximise engagement [4,5,6]. 

Because of longer periods spent in a sitting position, even the low-level physical activity of everyday life has been on the decline, leading to diminished physical fitness, as sitting is replacing a large proportion of day-to-day life, non-exercise physical activity that people had in former times [7]. Thus, there is considerable interest in analysing the potential role of low-intensity physical activities of everyday life in health strategies and in human susceptibility or resistance to weight gain [8,9,10,11]. In this context, there is growing interest in increasing EE by maintaining standing posture rather than sitting comfortably, but the impact of longer-duration standing postures on clinical outcomes in body fatness management are controversial [12,13,14]. To this end, the use of structured and unstructured low-intensity physical activities to increase EE is receiving more attention as a way to help weight regulation and to counteract the burden of sedentariness in healthy inactive subjects or those with chronic diseases [9,15]. 

Additionally, a low capacity for fat oxidation has often been implicated in the manifestation of chronic metabolic diseases and is associated with weight gain and co-morbidities [16,17]. As the relative contribution of fat oxidation rates to total EE has been consistently shown to be greater at low-to-moderate exercise intensities than during exercise at high intensities [18,19,20], exercises at low to moderate intensities are thus encouraged within physical activity programs to improve muscle oxidative function and fat utilisation ability in inactive individuals, and in particular, in those presenting cardiometabolic risks [21,22,23]. Hence, to favour both fat oxidation and subjects’ compliance, convenient and readily perceived as achievable physical activities set at appropriate intensities must be promoted, with the perception of physical exertion being defined as the subjective intensity of effort, discomfort, strain, and/or fatigue that one feels during exercising [24]. In this context, regarding the role for low-intensity physical activities in weight management, however, little is known about the behaviour of substrate oxidation in response to very light and light exercise as compared to being at rest (e.g., sitting inactive) [25]. In addition, while the rate of perceived exertion (RPE) appears to be an interesting tool to monitor physical activities and promote individuals’ adherence, there is a lack of data regarding the relationship between RPE and fat oxidation in the category of light physical activities [4,24,26].

The main objective of the present study, therefore, was to gain insights into the dynamics of EE and substrate oxidation from sitting comfortably (in the resting state) to very low and low intensity exercise and to determine the inter-relationships between the RPE, substrate oxidation, and EE during light-intensity cycling in inactive men and women.

## 2. Results

### 2.1. Experiment I: No-Load Cycling 

**Figure 1:**
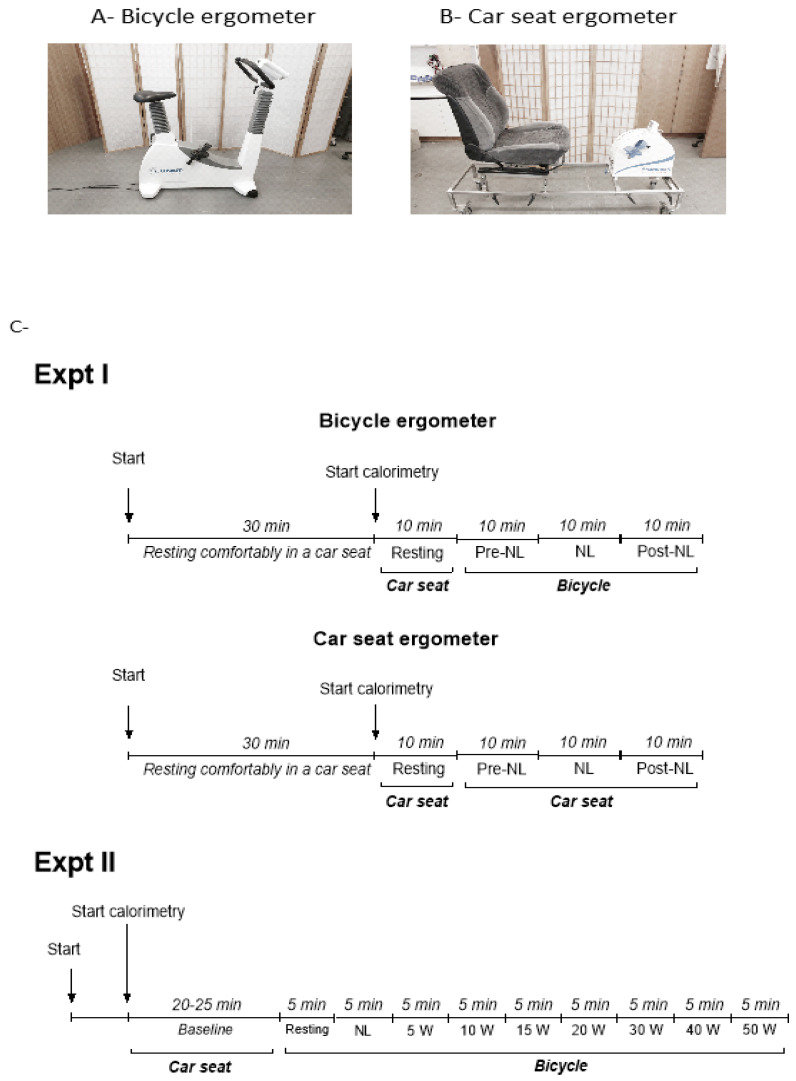
Photographs of the two types of cycling ergometry utilised (**A**,**B**) and experimental design illustration (**C**). NL = no-load cycling: sitting (car seat or bicycle) and cycling at 60 rpm at no-load.

Based upon previous studies [27,28] indicating that no-load cycling could lead to an increase in EE above rest by more than 50%, we investigated, in 16 subjects (8 men and 8 women), the impact of this minimal (no-load) cycling movement on both EE and respiratory quotient (RQ) using two cycling modalities: a bicycle ergometer and a car seat ergometer (Figure 1, panels A and B, respectively). In a previous validation study, we showed that after overnight fasting, the sitting EE of subjects in this ergonomic car seat was not different from supine EE, i.e., when compared to EE measured under conditions of basal metabolic rate assessment [29]. Experiment I was performed in two sequential phases in the morning, with a bicycle ergometry phase and a car seat ergometry phase separated by 30 min rest in the car seat; the sequence of these two phases (car seat ergometer or bicycle ergometer) was randomised across subjects; the design of this experiment I is depicted in Figure 1C (upper panel). 

*Panel A*: the bicycle ergometer (Cosmed E100 P, Milan Italy). *Panel B*: the car seat ergometer—i.e., a car seat which was mounted with a Monark ergometer (Rehab Trainer 881E, Monark Exercise AB, Vansbro, Sweden) on a rectangular metal frame on wheels with strong brakes. In this car seat ergometer, the standardised posture of the subject at rest during baseline measurements was to sit with the feet along the sides of the metal frame, and the angle of inclination of the seat’s back support was adjusted between 110° and 120° for the subject’s maximal comfort. Prior to the cycling exercise, the position of the Monark ergometer, which could slide horizontally along the metal frame, was adjusted for each subject such that when the subject’s feet were at rest in the ‘pedals’ of the ergometer, the angle between the subject’s thigh (femur) and lower leg (tibia) was a right angle (90°). *Panel C*: study designs—upper panel: Experiment I (Expt I; n = 16); lower panel: Experiment II (Expt II; n = 19).

**Figure 2:**
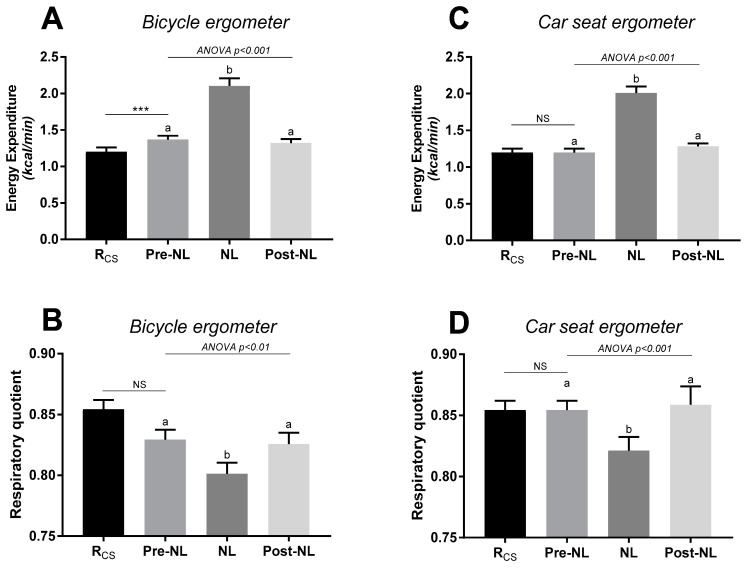
Energy expenditure (upper panels) and respiratory quotient (lower panels) at rest and in response to no-load (NL) cycling using the bicycle ergometer (panels **A**,**B**) or the car seat ergometer (panels **C**,**D**). Rcs = at rest while sitting in car seat ergometer; NL= no-load cycling: sitting (car seat or bicycle) and cycling at 60 rpm at no-load; pre-NL and post-NL: at rest while sitting in car seat or bicycle with feet on pedals before and after no-load cycling, respectively. Values are mean ± SEM. An ANOVA test was applied across pre-no-load, no-load, and post-no-load, followed by post hoc pairwise comparisons using Tukey’s test; values with different superscripts (a, b) are significantly different from each other (*p* < 0.05). A paired *t*-test was applied for comparing Pre-NL vs Rcs; ***: significant difference at *p* < 0.001.

The results of EE and RQ responses to no-load cycling are shown in Figure 2. Relative to sitting at rest in the ergonomic car seat, sitting at rest on the bicycle ergometer (without cycling) resulted in a small but significant increase in EE (+14.2%, *p* < 0.001) and a non-significant reduction in RQ (panels A and B, respectively). No-load cycling on the bicycle ergometer impacted significantly on both EE and RQ, namely increasing EE to reach ~1.75 times resting levels and decreasing RQ by 0.04 units (panel A and B); EE and RQ returned to their respective baseline (pre-no-load) levels in the subsequent post-no-load rest period (panels A and B). The same pattern of responses in EE and RQ was also observed when the test was performed entirely while seated in the ergometric car seat, with the no-load cycling increasing EE by 1.6 folds and decreasing RQ by 0.03 units, relative to sitting at rest in the car seat (panel C and D). 

**Figure 3:**
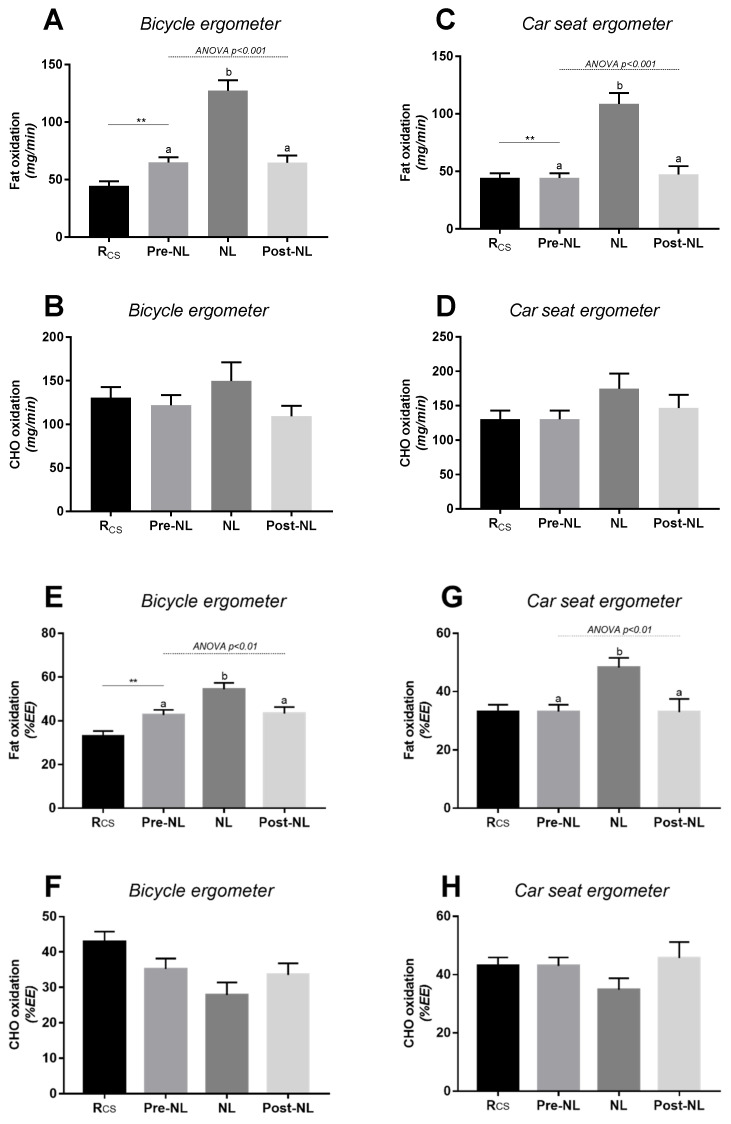
Substrate oxidation rates at rest and in response to no-load (NL) cycling using the bicycle ergometer (panels **A**,**B**) or the car seat ergometer (panels **C**,**D**). Rcs = at rest while sitting in car seat ergometer; NL = no-load cycling: sitting (car seat or bicycle) and cycling at 60 rpm at no-load; pre-NL and post-NL: at rest while sitting in car seat or bicycle with feet on pedals before and after no-load cycling, respectively; CHO: carbohydrates; EE: energy expenditure. Values are mean ± SEM. In Figure 3**A**–**D** the data of substrate oxidation are expressed as mg/min, while in Figure 3**E**–**H**, they are expressed as a percentage of energy expenditure (%EE). An ANOVA test was applied across pre-no-load, no-load, and post-no-load, followed by post hoc pairwise comparisons using Tukey’s test; values with different superscripts (a, b) are significantly different from each other (*p* < 0.05). A paired *t*-test was applied for comparing pre-NL vs Rcs; **: significant difference at *p* < 0.01.

The changes in RQ in response to no-load cycling were reflected primarily in changes in fat oxidation, with no significant changes in carbohydrate oxidation (Figure 3). Analysis of the data according to gender indicated a similar pattern of responses in EE, RQ, and substrate oxidation rates in response to no-load cycling (Supplementary Figures S1 and S2). 

### 2.2. Experiment II: Graded Cycling Exercise in Very Low to Low Power Range

To gain insights into the dynamics of substrate oxidation as a function of graded cycling exercise intensity and perceived exertion, experiment II, conducted in 19 subjects (7 men and 12 women), investigated the changes in EE, RQ, heart rate (HR), and RPE across very low-to low-intensity exercise on the bicycle ergometer across no-load to 50 W; namely 5 min cycling at no-load followed by 5 min at 5, 10, 15, 20, 30, 40, and 50 W, as indicated in the design depicted in Figure 1C (lower panel).

**Figure 4:**
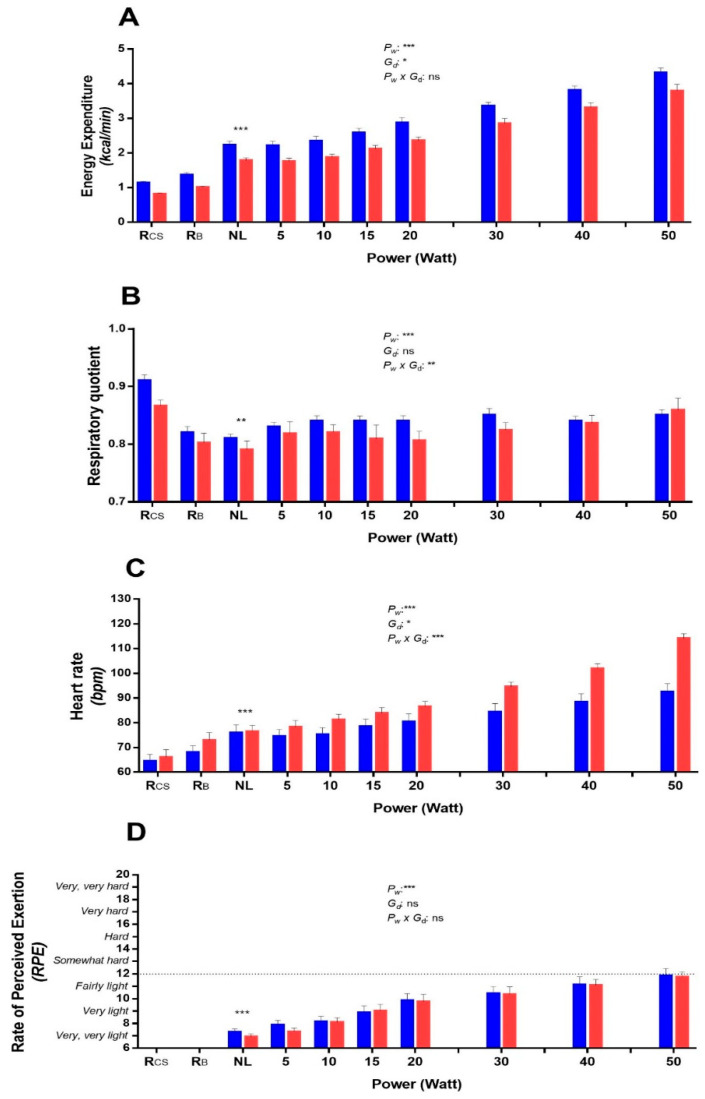
Effects of very low-to low-power cycling on energy expenditure (**A**), respiratory quotient (**B**), heart rate (**C**), and rate of perceived exertion (**D**) in men (in blue) and women (in red). Values are mean ± SEM. A paired *t*-test was applied for comparing no-load vs Rcs; **, ***: significant differences at *p* < 0.01 and *p* < 0.001, respectively, for both men and women. Repeated measure ANOVA was applied to the data across 5 to 50 watts to test for the effects of power (P*w*), gender (G*d*), or interaction (P*w* x G*d*); *, **, ***: significant differences at *p* < 0.05, *p* < 0.01, and *p* < 0.001, respectively; ns: non-significant. Rcs: resting energy expenditure in car seat (baseline); RB: resting energy expenditure while sitting inactively (no cycling) on the bicycle ergometer; NL = No-load cycling: sitting (bicycle) and cycling at 60 rpm at no-load.

The results of this experiment investigating the changes in EE, RQ, HR, and RPE across very low- to low-intensity cycling exercise on the bicycle ergometer are shown in Figure 4. In response to no-load cycling, EE increased by about 2 folds (*p* < 0.001), while RQ dropped (*p* < 0.01) in comparison to values during resting in the car seat (panels A and B, respectively). A linear increase in EE between 2–4 times above resting levels was observed across 5 to 50 W (*p* < 0.001), and although RQ increased significantly across this range (*p* < 0.001), this was very modest, and these RQ values across very low- to low-power cycling remained below that observed while at rest. Both HR and RPE increased linearly or curvi-linearly across 5 to 50 W (*p* < 0.001), but the mean RPE did not exceed values corresponding to the exercise being perceived as ‘light’ (panels C and D, respectively).

**Figure 5:**
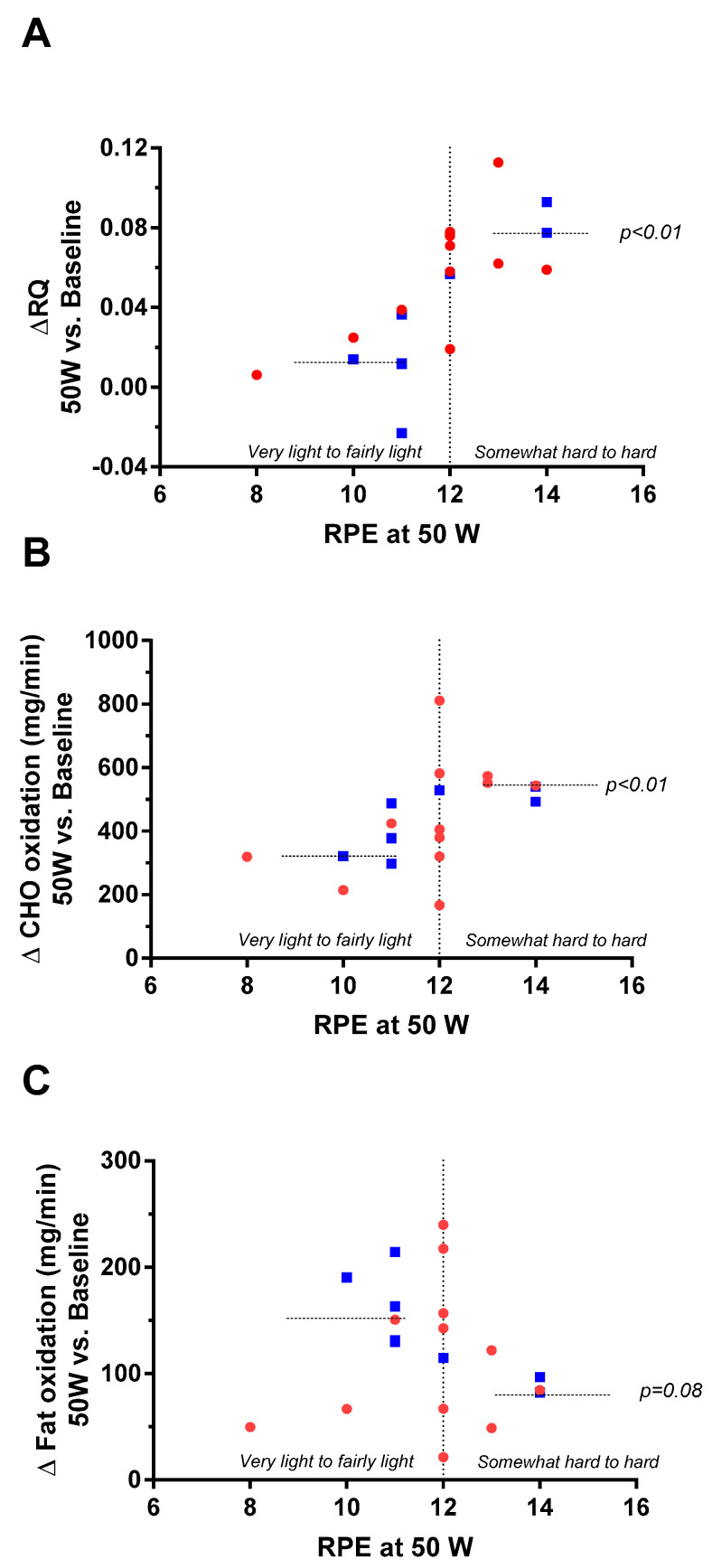
Plots of changes in respiratory quotient (**A**; Δ RQ), carbohydrate (**B**; Δ CHO) oxidation, and fat (**C**; Δ fat) oxidation at 50 W relative to baseline (RB) as a function of the rate of perceived exertion (RPE) at 50 W in men (in blue) and women (in red).

However, analysis of individual data of RPE at 50 W revealed two distinct clusters of subjects (Figure 5, panel A): those having RPE values corresponding to the exercise being perceived as ‘very light to fairly light’ (i.e., RPE < 12) and showing little or no increase in RQ relative to baseline resting levels, as opposed to an increase in RQ in those who perceived the exercise as being ‘somewhat hard to hard’ (i.e., RPE > 12); the difference in their changes in RQ being significant (0.01 vs. 0.08, *p* < 0.01 by test of median). The latter increase in RQ resulted primarily from significantly higher carbohydrate oxidation (*p* < 0.01, panel B) and a tendency for a lower fat oxidation (*p* = 0.08, panel C).

Regression analysis of the change in RQ at 50 W relative to baseline was also found to be positively associated with RPE at 50 W (r^2^ = 0.51, *p* < 0.001). However, neither RPE nor changes in RQ were found to be influenced by gender, height, body mass index (BMI), or body composition (fat mass, fat-free mass, body fat%).

**Figure 6:**
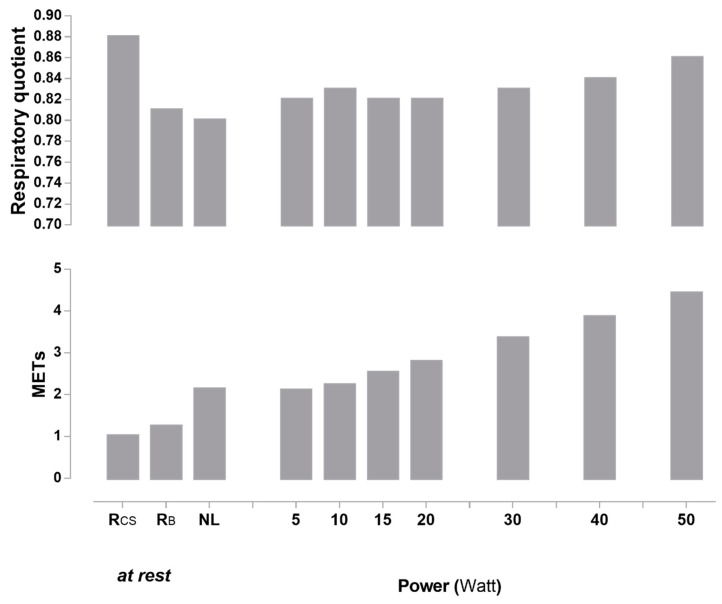
Respiratory quotient (upper panel) and METs (lower panel) at rest and in response to no-load (NL) cycling and across very low- to low-intensity cycling exercise. METs were calculated as the ratio of the work metabolic rate to the resting metabolic rate while sitting comfortably. Rcs: resting energy expenditure in car seat (baseline); RB: resting energy expenditure while sitting inactive (no cycling) on bicycle ergometer; NL = no-load cycling: sitting (bicycle) and cycling at 60 rpm at no-load; METs = metabolic equivalent of tasks.

Figure 6 depicts RQ (upper panel) and EE (expressed in METs - metabolic equivalent of tasks; lower panel) patterns in response to very low and low power cycling increments. While there was a strong linearity between power and EE between 5 to 50 W, the RQ in response to very light and light exercise followed more of a ‘U’ or ‘J’ shaped relationship.

## 3. Discussion

Although it is now recognised that low physical activity in daily life importantly contributes to variations in daily EE [11], there is scarce information regarding the impact of low-intensity physical activities on substrate oxidation relative to the resting state. In the study reported here, we showed that compared to comfortably sitting at rest, even very low-intensity leg cycling (<10 W) resulted in about a 2-fold increase in EE and a significant increase in preferential fat oxidation. Furthermore, the studies presented here showed that within the range of very low-to low-power cycling (5–50 W), the observed 2–4-fold increase in EE was associated with a relatively high fat oxidation rate, and that a shift to a preferential increase in carbohydrate oxidation seemed to occur with a change in perception of the exercise from being light to somewhat hard. 

### 3.1. Low Intensity vs. Sedentary Behaviour

In experiment I, the participants performed very light exercise (no-load cycling) on two different types of cycling equipment. Indeed, while bicycle ergometry is a common modality for cycling, the car seat ergometry used here highlighted advantages, as it did not involve posture modification from sedentary behaviour (i.e., sitting comfortably at rest) to the dynamics of leg cycling movement. Thus, the increase in EE and fat oxidation observed during the no-load cycling relative to sitting at rest could not be attributed to posture changes and the associated isometric work [27]. The car seat ergometer was also more comfortable and easier to propose for sessions lasting several hours or for persons not used to cycling or those with physical limitations (e.g., those with obesity, elderly persons) [29]. Indeed, EE was higher while sitting on the bicycle ergometer than while sitting in the car seat, likely because of the energy required for postural maintenance, which was more important on the bicycle than in a car seat where the trunk of a subject was supported [27]. 

Regarding substrate oxidation, it is well known that the contribution of carbohydrates and fat to fuel utilisation during exercise is dependent upon the intensity and the duration of exercise as well as genetics, age, gender, body composition, and nutritional and weight status [30,31]. Because the proportion of EE due to fat oxidation observed during low-to-moderate intensity physical activities has been shown to decrease when the exercise intensity rises [30], prolonged exercise at a low-to-moderate intensity is thus recommended to maximise fat utilisation and to prevent the development of metabolic disease [21,22,23,32]. However, there is scarce information about how substrate oxidation during light intensity exercises behaves compared to that in the resting state, with only one study reporting an increase in fat oxidation in response to low-intensity isometric exercise relative to being at rest [25]. In the present study, we show a significant impact of light-intensity cycling on substrate utilisation relative to sitting at rest. Specifically in response to the increased EE during no-load cycling, the decrease in RQ was primarily due to an increase in fat oxidation rates without a significant change in carbohydrate oxidation. Furthermore, RQ on average increased only slightly across very low- to low-intensity cycling (5–50 W), such that across such light intensity cycling, substrate oxidation remained in the zone of relatively high fat oxidation. 

According to the subjects’ RPE, it appeared that low-perceived cycling exercise (<3 METs) might have substantially increased EE and favoured fat oxidation rates relative to total EE. The present results suggest that relative to an inactive sitting position, sitting while leg cycling results in a preferential increase in fat oxidation during a dynamic exercise perceived as light. Other studies with various types of physical activities have previously reported significant changes in substrate responses to low-intensity exercises and their relevance in favouring fat oxidation [25,32,33,34]. In these studies, peak fat oxidation was reached during low-intensity exercise, or a higher fat oxidation rate was observed during low-intensity compared to high-intensity exercise [32,33,34] or, as in our study here, when comparing low-intensity exercise with sitting inactively [25]. Taken together, low-intensity exercises seem advisable to subjects not used to practicing physical activity or those with limited abilities as they are feasible, well-accepted (i.e., RPE), and offer relevant metabolic adaptations. 

### 3.2. Specific Response during No-Load Cycling

It seems relevant to look specifically at no-load cycling in a general context of physical activity promotion in inactive individuals. Considering the impact of no-load cycling on EE, it appears to be an attractive approach to substantially increase EE compared to sedentary behaviour (i.e., sitting) and thus to participate in human body weight regulation in inactive subjects. To our knowledge, no previous study has investigated the impact of no-load cycling on substrate oxidation, and this study is the first one to highlight a “metabolic shift” in substrate oxidation responses between pre-no-load, brief-no-load, and post-no-load cycling. While simply sitting on a bicycle involves some activity of the postural muscles [27], the increased rate of oxygen consumption from sitting to no-load cycling should be attributed to volitional muscle contraction with a predominance of fat as fuel for adenosine triphosphate resynthesis. Indeed, it is known that fat is the predominant substrate used during low-intensity isometric exercise performed intermittently [25], but to our knowledge, this is the first study to demonstrate that no-load cycling increases EE primarily by increasing fat oxidation when compared to being at rest. The localisation of the fat that is being channelled to increase fat oxidation remains to be determined. Due to the exercise bout duration, it may be hypothesised that it is too rapid for adipocyte lipolysis activity optimisation, and it is more likely coming from intramyocellular lipid stores. However, future studies are needed to confirm this hypothesis. 

The results seem to be more pronounced in women than in men, particularly when the data on fat oxidation are expressed as a proportion of EE (Supplementary Figure S2). This could underscore the gender difference in substrate metabolism during exercise, currently reported in the literature for different exercise modalities [35,36,37,38]. Different factors such as specific oestrogen levels [39], free fatty acid availability [40], total amount of fat mass, and adipose tissue distribution [41,42] may explain this sexual dimorphism. 

Overall, due to its effortless characteristics, no-load cycling may be an interesting and initial approach to be prescribed in inactive and/or pathological populations to substantially increase EE and relative fat oxidation to total EE compared to sedentary activity (e.g., sitting).

### 3.3. Metabolic Shift during Low Perceived Exertion

As we wanted to mimic the range of increased EE compatible with low-level physical activity intensities of daily life, it was important to remain in low-perceived cycling and not to exceed objectively low-to-moderate physical activity intensities (i.e., 6 METs) [43]. The EE measured across 10–50 W for men and women varied in the range of ~2–4.5 METs, which energetically corresponded to a low level of physical activity (including everyday life, spontaneous, and structured physical activities) [43,44]. 

Although it is worth noting that HR at rest and across the range of low-power cycling was higher in women compared to men, there was no gender difference in RPE at any workload. Indeed, in both men and women, the RPE on average did not exceed the value of 12 out of 20 on the Borg scale and were thus considered to be in the light zone [28]. Interestingly, the metabolic shift, namely a shift to increased carbohydrate oxidation rates, seemed to be associated with the subjects’ perception of the exercise difficulty, as revealed when examining inter-individual variability in RPE values at 50 W. It appeared thus relevant to determine if 50 W was a physiological and perceived threshold in light activity for fat oxidation rates in inactive subjects. Overall, while there was a strong linearity between power and EE between 5 to 50 W, the behaviour of substrate oxidation in response to very light and light exercise differed with the ‘linear’ increase in EE, with the RQ response following more of a ‘U’ or ‘J’ shaped relationship (Figure 6). It can be interpreted as follows: 

*- First*, the drop in RQ after the posture change from the car seat (Rcs) to the bicycle ergometer (RB), while in the resting state in both cases, may be explained by postural muscles burning more fat than carbohydrates to maintain the less-comfortable posture of sitting on the bicycle than in the car seat. 

*- Second*, at power outputs where all subjects perceived the exercise as very light to light (<50 W), RQ was relatively stable and stayed in the zone of relatively high fat oxidation as compared to sedentary behaviour (i.e., sitting). Changes occurred at powers where some subjects started to feel like the exercise was starting to become harder (40–50 W) and where EE exceeded the normal range of 2–3 METs in frequently fulfilled everyday tasks (such as sitting, standing, walking, and climbing stairs) [45]. 

*- Third*, the association observed between the RQ modification and the perception at 50 W showed a strong relationship between the change in perception from “fairly light” to “somewhat hard” and the shift from proportionally more carbohydrate than fat oxidation to sustain energy demand. These results emphasised the importance of maintaining even low-level physical activity rather than being sedentary for both the EE and the fat oxidation produced. 

Relative exercise intensity can be determined using a variety of parameters (e.g., % of maximal power output, % of maximal oxygen consumption, % of maximal HR, plasma lactate concentrations etc.). However, they are predominantly used in the context of sport training and performance and may not be appropriate and accurate regarding daily very low- to low-intensity activities. Moreover, they require laboratory investigations with specific and frequently expensive equipment and methods. The RPE appears as an interesting approach to estimate and monitor the intensity of exercise. While it remains a subjective method, it is a valid and affordable tool [46] considering the subject’s feelings, which seems essential in the perspective of long-term individual compliance to maximise physical activity engagement. 

The human energy balance is a complex phenomenon wherein the importance of physical activity in the matching of EE to energy intake is a fundamental feature. Very low- to low-intensity exercises have received more and more interest over the last decades, and the present findings highlight their potential role in increasing physical activity and fat metabolism relative to total EE. Our study reveals a relationship between the change in perception and the shift from fat to carbohydrate oxidation during very low- to low-intensity exercises, independently of gender and BMI. This could imply that as soon as one starts to experience that the exercise is no longer feeling ‘light’, the body starts to burn more and more carbohydrates proportionally to meet energy needs. This stresses the importance of maintaining at least a low level of physical activity rather than being sedentary to still be able to burn fat, as a low capacity to oxidise fat has been linked to obesity and comorbidities [47]. This does not mean that exercising at higher intensities is not important, as RQ only reflects the proportion of fat and carbohydrates that are being oxidised and not the absolute amounts, which are, of course, greater at higher intensities. 

It is worth noting that the exercises performed in the present study were weight bearing and characterised by a low level of perceived exertion. They could, thus, be an interesting and initial health management approach for inactive individuals, those with excessive sedentary time (e.g., tertiary employees), and/or disabled persons unaccustomed to exercise, favouring the subject’s compliance. Whether the present results are consistent findings amongst chronic patient cares warrants further study.

Although the findings of the present study are of importance to our understanding of the dynamics of substrate oxidation from resting to very low- and low-intensity cycling exercises, there are some limitations which should be considered in the interpretation of our results.

First, the sample size in each experiment was rather small, especially when considering gender dimorphism, and further studies with larger sample sizes are thus needed to confirm our results. Secondly, no biological and histological analyses associated with substrate metabolism were performed in the present study, which, hence, limited the strength of our conclusions, based solely on the assessment of whole-body substrate metabolism by indirect calorimetry. Future studies should consider merging whole-body indirect calorimetric studies with the assessment of blood hormones and metabolites (e.g., glucose, insulin, triglycerides, free fatty acids, glycerol, catecholamines, adipokines, fibroblast growth factor 21) as well as intra-myocellular lipid and glycogen content. Finally, the homogeneity of the present population sample (i.e., healthy young adults of European Caucasian descent) does not allow the generalisation of the results to other populations. Whether these findings are consistent amongst people of different ethnicities and/or populations with impaired metabolic status warrants further study.

## 4. Materials and Methods

### 4.1. Subjects 

For each experiment, the subjects were recruited by advertisements (placed at different signposts of the buildings) at our university and other higher educational institutes in Fribourg. The inclusion criteria were that the subjects were healthy young adults in the age range of 18–35 years, with BMI in the range of 18 to 29 kg·m^−2^, stable body weight (defined as <3% variation during the past 6 months), and who were not athletes or physically active. Smokers, pregnant or breast-feeding women, claustrophobic individuals, individuals taking medication, and those with any metabolic diseases were excluded. Due to menstrual cycle influences on energetic balance, eumenorrheic women were only tested during the follicular phase of their menstrual cycle [48].

Among the responders to the advertisement, all were students or young research staff living in the urban areas of the canton of Fribourg (Switzerland). After a first visit to our laboratory to confirm eligibility, they were all found to be eligible for the study according to the above-mentioned inclusion and exclusion criteria. In particular, the selection of subjects as non-physically active or “inactive” was ascertained through an interview and the completion of a questionnaire to determine their diet and lifestyle habits (including habitual physical activity, with a specific focus on time spent on moderate-to-vigorous aerobic physical activity). As previously described [28], the subjects were considered to be ‘inactive’ in accordance with the proposal of the Sedentary Behaviour Research Network [49] in referring to individuals who habitually do not perform sufficient amounts of moderate-to-vigorous aerobic physical activity, and who, in our study, did not meet the Canadian Physical Activity Guidelines [50], namely a minimum of 150 min of moderate-to-vigorous aerobic physical activity per week, in bouts of 10 min or more. None of the selected subjects were active cyclists, as they had no particular experience in cycling either for exercise training or as a mode of transportation. All participants completed the experimental protocol that was scheduled for a later date and took place in a single morning session in our laboratory, located in the Physiology building of the Department of Medicine, University of Fribourg. 

Overall, the study was conducted in healthy young adults (*n* = 35; 20 women) of European Caucasian descent, with their ages in the range of 19 to 32 years and their BMIs in the range of 18.1 to 27.2 kg·m^−2^. Specifically, 16 subjects (8 women) participated in experiment I, which was conducted from October to December 2018, and 19 subjects (12 women) participated in experiment II, which was conducted from March to July 2019. No subject participated in both experiments, and the general characteristics of the subjects involved in each experiment are provided in Table 1. No subject reported any ill effects during the experiment, nor afterwards in subsequent days and months later. 

All subjects gave their informed consent for inclusion before they participated in the study. The study was conducted in accordance with the Declaration of Helsinki, and the protocol was approved by the Cantonal Ethics Committee of Vaud (CER-VD 2017-02087).

As experiments I and II were exploratory studies about substrate oxidation in response to light cycling exercise, no previous data and evidence on this topic were available to conduct a priori sample size calculations. Yet, post hoc power analyses showed that to detect a 0.04 and 0.07 unit change in RQ (primary outcome) from resting to very low cycling, 16 and 19 subjects in each experiment, respectively, allowed the power detection to be >80%, with type-I error of 0.05.

### 4.2. General Study Design

For this exploratory study with a repeated measures design, all participants were requested to avoid moderate or vigorous physical activities, caffeine, and dietary supplements in the 24 h prior to testing. Body weight and height were measured twice using a mechanical column scale with an integrated stadiometer (Seca model 709, Hamburg, Germany), and BMI was calculated as body weight (kg) divided by height squared (m^2^). Body composition (fat mass, fat-free mass, and body fat%) was assessed by bioelectrical impedance analysis (Inbody 720, Biospace Co., Ltd., Seoul, Korea). On the test day, the subject arrived to the laboratory between 8:00 to 8:15 after an overnight fast of 10–12 h. Oxygen consumption (VO_2_) and carbon dioxide production (VCO_2_) were measured breath-by-breath through computer-assisted indirect calorimetry (Quark CPET Cosmed, Rome, Italy) using a Hans Rudolph silicon facemask in the following sequence: (i) for 20 to 30 min of rest comfortably seated in an ergonomic and adjustable car seat adapted for metabolic monitoring, (ii) for 10 min while sitting at rest in the car seat or on a bicycle ergometer, and (iii) during the subsequent cycling exercise. Values of EE and RQ were averaged over the last 5 min of the resting periods and over the last 2 min of cycling at each power output in the very low and low intensity ranges, namely during 5 min bouts of cycling at 60 rpm at no-load and across the range of 5 to 50 W. The RPE was assessed at the end of each trial using a validated perception scale. In order to prevent sleeping and reduce boredom and accompanying stress, participants were permitted to watch a calm movie or a documentary during the periods of rest. 

### 4.3. Measurements 

#### 4.3.1. Indirect Calorimetry and Heart Rate

Energy expenditure was calculated according to the Weir equation [51]: EE (kcal·min^−1^) = 5.68 VO_2_ (mL·min^−1^) + 1.59 VCO_2_ (mL·min^−1^) − 2.17 N_u_, where N_u_ was total urinary nitrogen excreted. As short-term urinary collections to assess total N_u_ might not be representative of the protein oxidised during the measurement itself, they were not obtained in this study and were assumed to be 13 g/24 h. This value reflected urinary nitrogen excretion of subjects in a fasted state [52], and as described previously, this assumption did not significantly influence the relative partition between carbohydrate and fat oxidation rates [25,53,54]. RQ was calculated as the ratio of VCO_2_ to VO_2_. Absolute carbohydrate and fat oxidation rates were calculated according to the following equations [55]:

Carbohydrate oxidation (mg·min^−1^) = 4.59 VCO_2_ (mL·min^−1^) − 3.25 VO_2_ (mL·min^−1^) − 3.68 N_u_ (mg·min^−1^)

Fat oxidation (mg·min^−1^) = 1.69 VO_2_ (mL·min^−1^) − 1.69 VCO_2_ (mL·min^−1^) − 1.72 N_u_ (mg·min^−1^)

Heart rate was continuously measured throughout the protocol by a wireless physiological monitoring system (Equivital EQ-01, Hidalgo, Cambridgeshire, UK). 

#### 4.3.2. Rate of Perceived Exertion

Prior to the tests, the range of perceived physical exertion that corresponded to effort categories within the Borg scale were explained to the participants to familiarise them with it. This scale was elaborated using a range of items from 6 to 20, with the number 6 corresponding to an extremely easy exercise, while an effort leading the participant to stop the test because of its hard difficulty was indicated by 20. At the end of each exercise bout, subjects were asked about their perceived exertion using the Borg scale [24,56].

### 4.4. Data and Statistical Analysis

All data are presented as mean ± standard error of the mean (SEM) unless otherwise stated, with a level of significance set up at *p* < 0.05. The Kolmogorov–Smirnov test was used to test the assumption of distribution normality for quantitative parameters. The statistical treatment of data by two way repeated-measures ANOVA followed by pairwise comparison tests (Tukey test), unpaired and paired *t*-tests, correlations, regressions, and median tests were performed using the computer software STATISTIX 8 (Analytical Software, St. Paul, MN, USA). 

## 5. Conclusions

Our study indicated that during very low- to low-intensity exercise, when EE was 2–4 times higher than at rest, RQ stayed in the zone of relatively high fat oxidation, while a shift from fat to carbohydrate oxidation was associated with a shift in perception from ‘light’ to ‘somewhat hard’. Given that compliance to exercise diminishes when it is perceived to be hard in inactive individuals, our study showing that high fat oxidation is maintained during ‘light-perceived’ physical activity reinforces further the importance of light physical activity in the prevention of obesity. 

## Figures and Tables

**Table 1:** General characteristics of the participants in experiments I and II.

Experiment	Variables	All subjects	Men	Women
**I**	**n**	16	8	8
	**Age (y)**	23.4 ± 0.8	24.5 ± 1.3	22.4 ± 0.8
	**Height (cm)**	173.4 ± 3.1	183.0 ± 2.65	163.8 ± 2.53 ***
	**Weight (kg)**	70.3 ± 3.9	83.7 ± 2.8	56.8 ± 2.6 ***
	**BMI (kg·m^−2^)**	23.1 ± 0.7	25.0 ± 0.7	21.1 ± 0.5 ***
	**FM (%)**	20.3 ± 1.7	16.9 ± 2.5	23.7 ± 1.8 *
	**FFM (kg)**	58.2 ± 4.4	73.7 ± 3.3	42.6 ± 1.9 ***
**II**	**n**	19	7	12
	**Age (y)**	24.9 ± 0.6	25.9 ± 1.1	24.3 ± 0.8
	**Height (cm)**	170.8 ± 1.8	177.7 ± 2.2	166.7 ± 1.6 ***
	**Weight (kg)**	64.6 ± 2.6	74.0 ± 4.7	59.1 ± 1.6 ***
	**BMI (kg·m^−2^)**	22.0 ± 0.5	23.3 ± 0.9	21.3 ± 0.6 *
	**FM (%)**	21.4 ± 1.8	15.8 ± 2.7	24.7 ± 1.8 **
	**FFM (kg)**	50.7 ± 2.3	61.8 ± 2.7	44.3 ± 1.1 ***

Mean ± SEM. BMI: body mass index; FFM: fat-free mass; FM: fat mass. *, **, ***: significantly different from men at *p* < 0.05, *p* < 0.01, and *p* < 0.001, respectively.

## Data Availability

The data presented in this study are available on request from the corresponding author. The data are not publicly available due to ethical.

## Supplementary Materials

The following are available online at https://www.mdpi.com/article/10.3390/metabo11060334/s1, Two supplementary figures are provided, Supplementary Figure S1: Energy expenditure (upper panels) and respiratory quotient (lower panels) at rest and in response to no-load (NL) cycling using the bicycle ergometer (panels A and B) or the car seat ergometer (panels C and D) in men (in blue) and women (in red), Supplementary Figure S2: Substrate oxidation rates at rest and in response to no-load (NL) cycling using the bicycle ergometer (panels A, B, E and BF or the car seat ergometer (panels C, D, G and H) in men (in blue) and women (in red).
