# Supplementary material for: Dynamics of Fat Oxidation from Sitting at Rest to Light Exercise in Inactive Young Humans
Source: Metabolites. 2021 May 24;11(6):334. doi: 10.3390/metabo11060334 (PMC8225068; doi:10.3390/metabo11060334)
Supplement: Supplementary file 1 [file metabolites-11-00334-s001.zip › metabolites-1177586-supplementary.pdf]

Supplementary Materials

Supplementary Figure S1

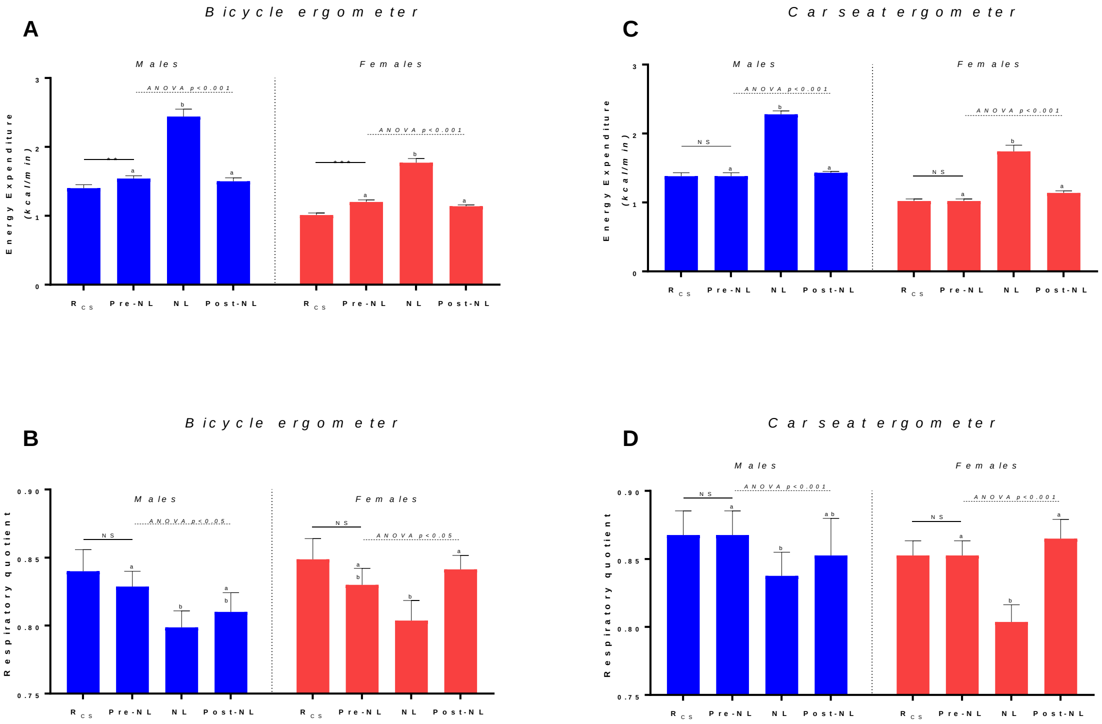

Supplementary Figure S1. Energy expenditure (upper panels) and respiratory quotient (lower panels) at rest and in response to no-load (NL) cycling using the bicycle ergometer (panels A and B) or the car seat ergometer (panels C and D) in men (in blue) and women (in red). Rcs = at rest while sitting in car seat ergometer; NL= No-load cycling: sitting (car seat or bicycle) and cycling at 60 rpm at no-load; Pre-NL and Post-NL = at rest while sitting in car seat or bicycle with feet on pedals before and after no-load cycling, respectively.

Values are mean ± SEM.

ANOVA test was applied across pre-no-load, no-load and post-no-load, followed by post-hoc pairwise comparisons using Tukey's test; values with different superscripts (a, b) are significantly different from each other ($p<0.05$).

Paired t-test was applied for comparing Pre-NL vs Rcs; **, ***: significant difference at $p<0.01$ and $p<0.001$, respectively.

Supplementary Figure S2

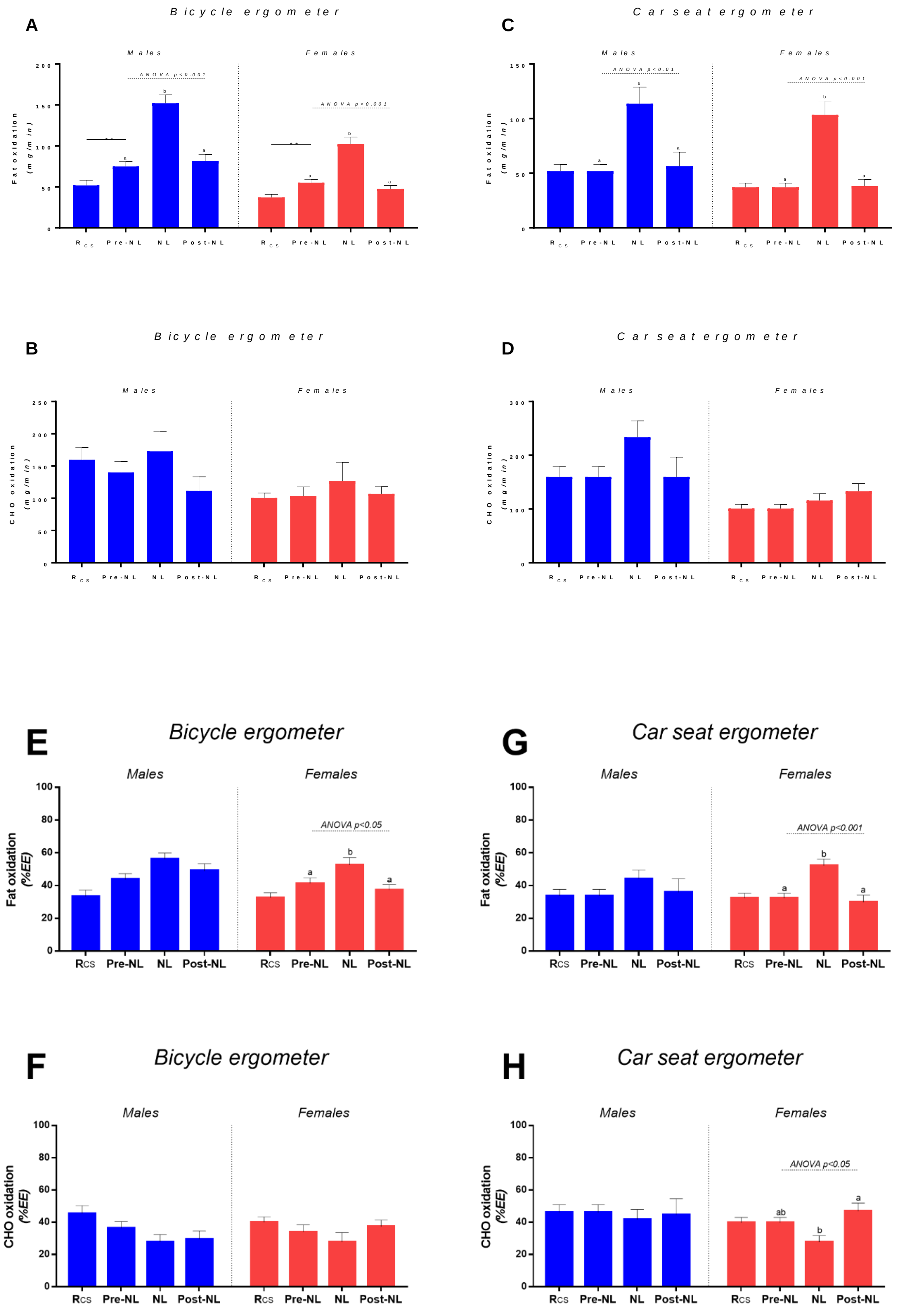

Supplementary Figure S2. Substrate oxidation rates at rest and in response to no-load (NL) cycling using the bicycle ergometer (panels A, B, E and F) or the car seat ergometer (panels C, D, G and H) in men (in blue) and women (in red). Rcs = at rest while sitting in car seat ergometer; NL= No-load cycling: sitting (car seat or bicycle) and cycling at 60 rpm at no-load; Pre-NL and Post-NL = at rest while sitting in car seat or bicycle with feet on pedals before and after no-load cycling, respectively; CHO = carbohydrates; EE = energy expenditure.

Values are mean ± SEM. In Figure S2 A, B, C and D, the data of substrate oxidation are expressed as mg/min, while in Figure S2 E, F, G and H, they are expressed as a percentage of energy expenditure (%EE).

ANOVA test was applied across pre-no-load, no-load and post-no-load, followed by post-hoc pairwise comparisons using Tukey's test; values with different superscripts (a, b) are significantly different from each other ($p<0.05$).

Paired t-test was applied for comparing Pre-NL vs Rcs; **: significant difference at $p<0.01$.
